# Serious games vs. traditional tutorials in the pandemic: a randomised controlled trial

**DOI:** 10.3389/fmed.2024.1424024

**Published:** 2024-11-21

**Authors:** Su Min Joyce Tan, Michael J. Coffey, Katrina Blazek, Neela Sitaram, Isabella Dobrescu, Alberto Motta, Sandra Chuang, Chee Y. Ooi

**Affiliations:** ^1^Department of General Paediatrics, Sydney Children’s Hospital, Sydney, NSW, Australia; ^2^Discipline of Paediatrics and Child Health, School of Clinical Medicine, Faculty of Medicine and Health, University of New South Wales, Sydney, NSW, Australia; ^3^Department of Gastroenterology, Sydney Children’s Hospital, Randwick, NSW, Australia; ^4^School of Population Health, Faculty of Medicine and Health, University of New South Wales, Sydney, NSW, Australia; ^5^School of Economics, UNSW Business School, University of New South Wales Sydney, Sydney, NSW, Australia; ^6^Department of Respiratory Medicine, Sydney Children’s Hospital, Randwick, NSW, Australia

**Keywords:** serious games, game-based learning, virtual, tutorial, medical education

## Abstract

**Background and aim:**

The COVID-19 pandemic necessitated the transition to online medical education. This study evaluated the efficacy of online case-based tutorials using a serious game tutorial [PlayMed™ (PM)], as compared to a traditional slideshow tutorial (TT).

**Methods:**

We performed a prospective, mixed-methods, randomised controlled trial on undergraduate medical students during the COVID-19 pandemic, from May 2020 to January 2021. Students were block randomised into the PM or TT groups. Tutors conducted online teaching on bronchiolitis and gastroenteritis cases using PM or TT to facilitate the presentation. Educational experience was assessed using a continuous interval scale (0–100; with pre-defined categories) and free text responses. Immediate and long-term knowledge acquisition was assessed using 6 multiple-choice questions (MCQ) for each case (total of 12 MCQ). A modified intention-to-treat mixed methods and a sensitivity per-protocol analysis were performed to compare outcomes between PM and TT groups.

**Results:**

In total, 80 PM and 73 TT participants attended at least one tutorial. Sixty-five (81%) PM and 52 (71%) TT participants completed at least one survey and were included for analysis. PlayMed™ students had an increased likelihood of completing the surveys, which included the MCQ [odds ratio (95% CI) of 2.4 (1.6–3.8), *p* < 0.00006]. Regarding the immediate reactions post bronchiolitis and gastroenteritis cases, several responses were significantly more positive in the PM group compared to the TT group; e.g. ‘The learning activity was engaging’ [medium effect size: *d* (95% CI) = 0.58 (0.32–0.85), *p* < 0.0001]. Higher proportions of participants in the PM group reported feeling safe in the gastroenteritis and bronchiolitis tutorials (96 and 89%), compared to the TT group (76 and 74%). PlayMed™ participants significantly outperformed TT participants on the bronchiolitis MCQs done immediately post tutorial, 4.1 (1.0) vs. 3.5 (1.0), respectively, *p* = 0.004 [medium effect size: *d* (95% CI) = 0.54 (0.16–0.91)].

**Conclusion:**

This study demonstrates the utility of a serious game (PlayMed™) as an online teaching tool for medical education. Students exposed to PM demonstrated superior engagement and feelings of safety. Utilisation of serious games may also facilitate knowledge acquisition, at least in the short term.

## 1 Introduction

In recent years, hastened by the COVID-19 pandemic, digital and information technology have transformed society and various sectors in life including delivery of medical education and clinical care. During the pandemic, particularly in regions where lockdowns were implemented strictly, medical teaching moved online while face-to-face and bedside clinical teaching were heavily restricted (1, 2).

Traditional case-based tutorials are widely used in online medical education. These typically use clinical cases and scenarios to explore learning objectives of the curriculum such as generation of differential diagnoses, using investigations to support or refute differentials and development of clinical management plans. For online traditional case-based tutorials, tutors and students meet over virtual platforms. A slideshow presentation is commonly used by tutors. Students participate via live sharing of questions, answers and comments via microphone audio or chat functions. Immersive technologies and virtual medical education continues to play an important role in medical education with key benefits in accessibility, flexibility, cost-effectiveness and standardisation (3).

Serious games are educational tools, typically online, that are aimed to supply users with skills, knowledge, or attitudes useful in reality (4) without the high resource demands associated with traditional classroom or face-to-face teaching environments (5, 6). We had previously developed a highly-immersive serious game for paediatric medical education called PlayMed™ (PM) (7). PlayMed™ provided a unique platform which allowed the application Knowles’ theory of andragogy to facilitate adult learnings exploring and solving real-world problems (7). This theory provides a framework for adult learning by focusing on the principles of autonomy, self-direction and evaluation, and experiential and problem-based learning in a simulated workplace (8–10). Additionally, Kolb’s experiential learning theory provides a framework for understand and implementing experiential learning to allow learners to progress through the four stages in sequence: concrete experience, reflective observation, abstract conceptualisation and active experimentation (11). Online serious games have the benefit of immediate in-game feedback and repeatability in a safe environment that facilitate the application of Kolb’s experiential learning theory (11, 12). PlayMed™ was developed using multiple game design theories including, self-determination theory (13), MDA framework (14), flow theory (15), Raph Koster’s theory of fun (16) and importantly fiero and failure (17). PlayMed™ facilitates the integration of game and learning theories to provide an innovative and emotive environment. Players are faced with the meaningful challenge of managing virtual patients, a form of ‘Hard Fun’ which creates emotion through a structured and safe experience (17). Difficult cases can be frustrating and make require multiple attempts before succeeding, however the sense of personal triumph (i.e., Fiero) upon completion can be very rewarding and memorable. Additionally, the difficulty level of cases increases as the player progresses through the simpler cases, thus balancing game difficulty with the anticipated skill level to keep players engaged.

Perron et al. performed an investigator-blinded randomised controlled trial comparing learner attitudes and the educational efficacy of a serious game (PlayMed™) compared to both an adaptive tutorial (computerised, interactive, web-based lessons using the SmartSparrowTM platform) and a low stimulus control (published paper-based guidelines). Medical students were taught paediatric asthma and seizure assessment and management using one of the three interventions, and then assessed using multiple choice questions and simulated clinical management scenarios in a high-fidelity simulation laboratory. Students allocated to the serious game intervention demonstrated improved translation of knowledge in the simulated clinical scenarios, particularly compared to the low stimulus control (7).

The aim of our study was to assess if using a serious game as an online teaching tool by the tutor in medical student case-based tutorials [PlayMed™ (PM) group] was more effective than traditional slideshow and case-based online tutorials (TT) group. The primary aim was to evaluate and compare the student educational experience between the PM and TT groups, including assessment of student satisfaction, engagement and understanding, through surveys. The secondary aim was to evaluate and compare student learning and knowledge acquisition, assessing immediate and long-term retention of knowledge using multiple choice questions (MCQs). We hypothesised that the PM group would have better educational experience, learning and knowledge acquisition compared to the TT group.

## 2 Materials and methods

This study was approved by the University of New South Wales Human Research Ethics Committee (HC17160).

### 2.1 Study design

We performed a prospective, mixed-methods, randomised controlled trial (RCT) on undergraduate medical students attending the Faculty of Medicine at University of New South Wales, Sydney, Australia (UNSW) during the COVID-19 pandemic, from May 2020 to January 2021. This study was designed to assess the efficacy and educational experience of serious game enhanced case-based tutorials (PM group) compared with traditional slideshow case-based tutorials (TT group).

### 2.2 Participants

Phase 3 medical students (final years of Years 5 and 6) at UNSW who were enrolled in the Children’s Health course were eligible. The Children’s Health course is an 8-week core course delivered by the School of Women’s and Children’s Health (SWCH) and completed by metropolitan and rural students during the final 2 years (Phase 3) of the 6-year undergraduate medical programme. There are five course offerings annually with 45–75 students enrolled in each teaching period. Due to the COVID-19 pandemic and strict lockdown measures in place across Australia, the Children’s Health course lectures and tutorials were mostly delivered online with significantly reduced face-to-face student-patient clinical experiences.

One of the study investigators emailed students with information about the study, including the Participant Information Statement, at least 2 weeks prior to starting the Children’s Heath course. Students were informed that apart from additional learning opportunities provided by the tutorials, there were no financial or additional incentives to participate in the trial. Participation in the study was voluntary. Informed consent was obtained from the students.

### 2.3 Randomisation

All recruited students were randomly allocated to one of two online tutorial groups: PlayMed™ case-based tutorial (PM group), or traditional slideshow case-based tutorial (TT group). A computer-generated block randomisation list, with block sizes of 4 and 6, and a strata for teaching period was utilised.^1^ Students were allocated a unique identification number which was recorded against their student number and group allocation in a secure (password-protected) electronic database on UNSW SWCH drive. This was done by one of the SWCH Education Support Officers.

### 2.4 Learning content

The clinical assessment, diagnosis and management of bronchiolitis and acute gastroenteritis were selected as the focus of learning for the two online tutorials given to each PM or TT group. These two conditions are both addressed in the Children’s Health lecture programme and are frequently encountered in clinical rotations and clerkships. Importantly, acute gastroenteritis and respiratory infections like bronchiolitis are common paediatric emergency presentations in Australasia (18) and junior doctors will likely be involved in management. Australian clinical practise guidelines are freely available on the acute management of bronchiolitis (19) and management of acute gastroenteritis in infants and children (20). The key learning outcomes and case stems for these tutorials were determined using the Children’s Health course objectives and were the same for the PM and TT groups. The MCQs were written by paediatric doctors and reviewed by UNSW Medicine faculty educators to ensure that they were based on the same key learning outcomes.

### 2.5 Intervention

A single tutor developed and presented all the PM tutorials and a separate single tutor developed and presented all the TT tutorials. Each tutorial was limited to 45 min duration and delivered virtually via a cloud-based video conferencing platform, with students given the option of leaving their webcam turned on or off. Each tutor was a general paediatric fellow (in advanced specialist training for paediatrics) in the Sydney Children Hospital Network with similar level of paediatric training and medical student teaching experience. Both tutors were instructed to develop content based on the Australian clinical practise guidelines for the acute management of bronchiolitis (19) and the management of acute gastroenteritis in infants and children (20). Each tutor was blinded to the tutorial content of the other group and MCQs.

#### 2.5.1 PlayMed™ tutorial

**Figure 1:**
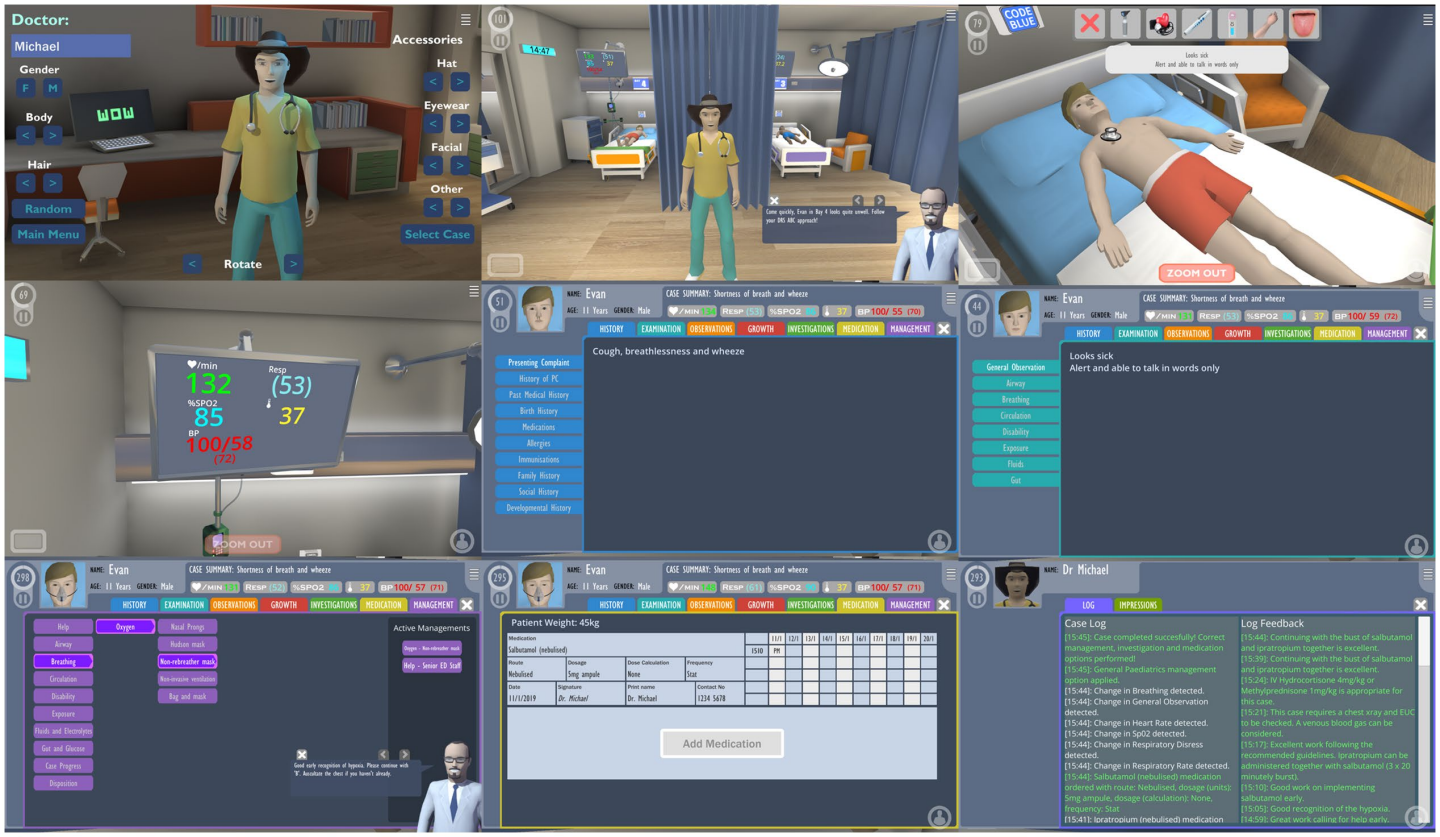
Various screenshots of PlayMed including: avatar customisation, gameplay in the hospital, bedside examination, review of vital signs, history and physical examination, management plan, medication prescription and in-game feedback log.

PlayMed™ [developed using the Playconomics™ platform (21) is a highly immersive role-playing game set in a virtual hospital with the player acting as a doctor (7)] (Figure 1; Supplementary Figure 1). It was developed using the principles of Knowles’ theory of andragogy (8) and Kolb’s experiential learning theory (11). Gameplay involves a player first creating a customisable avatar which is able to walk around the virtual hospital. Players then select a case to play, with each case involving a single patient that requires assessment and management. Players must attend to the bedside of the patient and perform an assessment within a timely fashion. The player is required to order appropriate investigations, prescribe medications and make appropriate management decisions using the in-game electronic medical record system. Patient status, vital signs and investigations change in real time and are dependent on the decisions made by the player. During the entire case, a simulated ‘attending physician’ provides real-time feedback on the player’s actions. The case is completed once the player has satisfactorily managed the patient. If a player makes inappropriate decisions, they are redirected by the attending physician or can even fail the case. Individual patient cases within the game were designed with complex case-flow algorithms (allowing for different combinations and permutations) and offered the opportunity for replay (7). A typical case would take around 5 to 7 min to complete, with additional feedback and performance rating (out of 5 stars) provided at the end.

In our study, a case on bronchiolitis and a second case on acute gastroenteritis were designed with complex case-flow algorithms, allowing for different combinations and permutations. The tutor acted as a single proxy player for the students by taking majority consensus suggestions on management from the student audience. Group polls were created using a free web-based poll maker and inserted at key decision points in the game. The majority student vote determined which in-game option was selected, e.g., choice of investigation or treatment option. In addition to real time tutor facilitation, an in-game simulated ‘attending physician’ provided real time feedback. For example, the majority of students may select to apply high flow nasal prongs in an infant with increase work of breathing and hypoxia. The virtual patient’s vital signs (i.e., respiratory rate and oxygen saturation) and visible work of breathing would then improve in the game.

#### 2.5.2 Traditional tutorial

The tutor delivered this using an online Microsoft PowerPoint slideshow presentation. The tutor went through slides sequentially, outlining the case scenario and teaching content was focused on the learning outcomes of the curriculum, while being flexible to students’ level of knowledge and clinical needs. The tutor of the TT encouraged students to ask and also answer questions throughout the tutorial. The tutor would facilitate these interactions periodically throughout the tutorial.

An Education Support Officer from SWCH emailed the students individually their unique identification number as well as the date, time, and Microsoft Teams access link to their case tutorial sessions. All tutorials (PM or TT) were delivered live using Microsoft Teams and an instruction sheet for students was provided on how to access the tutorials. The bronchiolitis and gastroenteritis tutorials were given in Week 1–2 of the 8-week Children’s Health course. Where possible, the PM and TT groups had their tutorials given simultaneously by their respective tutors except on one occasion where due to tutor sickness, the TT group had their tutorials rescheduled within 48 h. Immediately after each tutorial (PM or TT), and in Week 8 (last week of the Children’s Health Course), participants were invited to give feedback about their educational experience and have their knowledge assessed as outlined below.

### 2.6 Outcome measures

#### 2.6.1 Primary outcomes

Primary outcomes included a mixed methods analysis of the educational experience of participants, i.e., assessment of satisfaction, engagement and understanding. The students were invited to anonymously complete an online survey using Qualtrics immediately following each tutorial (Qualtrics, Provo, UT, United States). The immediate educational experience response survey is presented in Appendix 1A (Supplementary material). In the survey, students were asked to rate their responses to items on a continuous interval scale (0 to 100) with pre-defined categories: 1–20: Strongly Disagree; 21–40: Disagree; 41–60: Neutral; 61–80: Agree; 81–100: Strongly Agree; 0: No response. Students were also asked for comments (free text responses) regarding their engagement with, and perception of the educational value, of the teaching tool. The time commitment required of participants for each assessment was approximately 5 min.

#### 2.6.2 Secondary outcomes

A second survey was completed at Week 8 post tutorials to assess the long-term educational experience response (at the conclusion of the Children’s Health Course). This survey aimed to capture the follow-up impact of the tutorials on the student’s learning (Appendix 1B, Supplementary material).

Additional secondary outcomes were the immediate knowledge acquisition and long-term retention of knowledge, assessed using MCQs. All MCQs were completed online using Qualtrics. For immediate knowledge acquisition, participants were assessed immediately after the tutorial (PM or TT) using 6 case-specific MCQs for the bronchiolitis tutorial and 6 case-specific MCQs for the gastroenteritis tutorial (Appendices 2A,B, Supplementary material). The time commitment required of participants for each assessment was approximately 15 min. For long-term retention of knowledge, participants were assessed in a single sitting in Week 8 of the course using the same MCQ questions as before (total of 12 MCQs; six MCQs for bronchiolitis and six MCQs for gastroenteritis). The time commitment required of participants for this assessment was approximately 30 min.

### 2.7 Data collection

Participant information was collected in a series of electronic data collection forms with the Qualtrics Online Survey Software Tool using the study ID number as the identifier. The surveys were programmed to facilitate a personalised flow depending on the stage of the trial each participant was at, i.e., immediately post intervention (post-tutorial) or end of Children’s Health course (week 8). Post-tutorial and end of course surveys and MCQs were embedded in the survey. Email links were securely emailed to participants by an Education Support Officer from SWCH.

All study investigators were blinded to the assessment scores and feedback from the surveys.

### 2.8 Sample size calculation

The required sample size for this study was calculated using the following conditions: (i) based on data from our previous randomised controlled trial (7), 38.9% of serious game (i.e., PlayMed™) and 16.7% of adaptive tutorial (using Smart Sparrow) students ‘strongly agreed’ that the ‘activity provided helped me prepare for, and deal with, real life clinical scenarios’; (ii) relative group sizes of 1:1 for PM and TT; (iii) the sample size is calculated to detect a difference in incidence of the dichotomous outcome (‘strongly agree’ vs. any other response) between the two groups using the results above; (iv) type 1 error probability of 0.05 (two-tailed); (v) desired power of 0.8; and (vi) the ClinCalc Sample Size Calculator (https://clincalc.com/stats/samplesize.aspx) was used (22).

Based on these calculations, a minimum sample size of 63 PM and 63 TT students was required to reject the null hypothesis that the incidence of a ‘strongly agree’ response in the experimental (PM) and placebo-controlled (TT) groups were equal with probability (power) 0.8 and Type I error of 0.05. Assuming a total refusal and drop-out rate of 10%, 70 subjects for each cohort were required, i.e., 70 PM and 70 TT students.

### 2.9 Statistical analysis

Statistical analysis of the quantitative data was performed using descriptive and inferential statistics, calculated according to normality of distribution. Normally distributed data is presented by mean and standard deviation (SD) and non-normally distributed data was presented by median and interquartile range (IQR). Quantitative data was analysed on a modified intention-to-treat (mITT) basis with participants who answered at least one survey included. Missing quantitative data from the surveys and MCQs immediately post the tutorials (Appendix 1A, 2A,B, Supplementary material) were estimated by group mean imputation using the MICE package (v3.16.0) (23). A sensitivity per protocol analysis was also performed with missing quantitative data treated as missing. Given more than 50% of the data from the week 8 survey and MCQs (Appendix 1B, 2A,B, Supplementary material) was missing from both groups, only a per protocol analysis was performed.

Cross-sectional group comparisons (TT vs. PM) were made using quantitative data and performing an unpaired t-test or a Mann–Whitney test for parametric and non-parametric data, respectively. A chi-square test was used to compare categorical data between groups. *p* values <0.05 were considered statistically significant for all analyses. Effect sizes were calculated for statistically significant variables using Cohen’s *d* (*d*) with 95% confidence intervals (95% CI) using the ‘effsize’ package. Effect sizes were considered small if 0.2 ≤ *d* < 0.5, medium if 0.5 ≤ *d* < 0.8, and large if *d* ≥ 0.8. All quantitative analyses were performed by author MC who was blinded to the group allocation.

Qualitative data from open-ended survey questions were coded using a thematic, inductive approach by author KB who could not feasibly be blinded to group allocation. Data was manually transcribed into NVIVO ™ which assisted with data immersion. Labels were created and assigned to text, which described key meanings and ideas. After the first pass, and with each successive pass, codes were subsequently refined by aggregating common key themes and ideas. Group consensus was sought in cases where the meaning of the response was unclear. The proportion of responses related to each code were compared between tutorial type.

Given the difference in survey response rates between the TT and PM groups, a logistic regression analysis was performed. To determine the likelihood of completing the survey, a conditional logistic regression was performed using a dummy variable for MCQ completion (1 = yes; 0 = no). NVivo 1.7.1, R v4.2.0 and RStudio 2023.09.1 were used for the statistical analyses.

## 3 Results

### 3.1 Demographics and survey responses

A total of 153 students attended at least one of the online tutorials, consisting of 73 students in the TT group and 80 students in the PM group. Seventy-one percent (52/73) of the students in the TT group and 81% (65/80) of the students in the PM group provided responses to one or more surveys (immediately post tutorial or at the end of the Children’s Health course) and were included in the analysis (Table 1). The TT group had a significantly lower response rate in the post gastroenteritis tutorial survey compared with the PM group (54% vs. 83%, respectively, *p* = 0.001). Overall, PM students had an increased likelihood of completing the surveys, which included the MCQ [odds ratio (95% CI) of 2.4 (1.6–3.8), *p* < 0.00006].

**Table 1:** Demographics and survey responses.

Characteristics	Traditional tutorial	PlayMed™ tutorial
Participants, *n*	52	65
Age, mean years (SD)	23.1 (1.4)	23.3 (1.9)
Gender		
Male, *n* (%)	19 (37%)	26 (40%)
Female, *n* (%)	33 (63%)	38 (58%)
Not specified, *n* (%)	0 (0%)	1 (2%)
Year		
5, *n* (%)	42 (81%)	53 (82%)
6, *n* (%)	10 (19%)	12 (18%)
Completed surveys		
Post bronchiolitis tutorial, *n* (%)	44 (85%)	58 (89%)
Post gastroenteritis tutorial, *n* (%)	28 (54%)	54 (83%)
8 weeks, *n* (%)	11 (21%)	25 (38%)

Demographic data for the included participants is presented in Table 1. There were no significant differences between both groups for demographic variables of age, sex, or year.

Survey completion rates are presented in Table 1.

### 3.2 Primary outcome measures

The primary aim was to evaluate and compare the educational experience of both groups immediately post the bronchiolitis and gastroenteritis tutorials.

#### 3.2.1 Immediate reactions to the educational experience: quantitative analysis

A mITT analysis of the immediate reactions to the bronchiolitis and gastroenteritis cases is presented in Table 2.

**Table 2:** Immediate reactions to the educational experience (mITT analysis).

	Traditional tutorial	PlayMed™ tutorial	*p* value	*d* (95% CI)
**Combined responses post bronchiolitis and gastroenteritis cases, *n***	104	130		
I enjoyed the learning activity.	69.6 (18.8)	75.7 (16.5)	0.01*	0.35 (0.08–0.61)
The learning activity was engaging.	68.7 (19.4)	78.9 (16.0)	<0.0001*	0.58 (0.32–0.85)
I was able to understand the content delivered during the learning activity.	82.9 (14.4)	79.7 (15.6)	0.1	
I believe this learning activity will be beneficial for my paediatrics term.	79.2 (15.8)	80.4 (14.7)	0.6	
The learning activity will help me prepare for and deal with real-life clinical scenarios.	74.9 (14.9)	75.2 (15.0)	0.9	
I would attend a similar learning activity in the future.	79.8 (17.0)	79.0 (18.7)	0.7	
I would recommend this learning activity to a colleague.	74.5 (18.7)	77.3 (19.3)	0.1	
Receiving feedback during the learning activity aided my learning.	67.1 (21.0)	77.4 (18.5)	0.0001*	0.52 (0.26–0.78)
Your learning activity was more engaging than face-to-face teaching.	43.6 (21.0)	63.9 (23.8)	<0.0001*	0.90 (0.63–1.17)
**Bronchiolitis case responses, *n***	52	65		
I understand how to assess and manage a child with bronchiolitis.				
Before tutorial	46.8 (19.1)	42.8 (19.6)	0.3	
After tutorial	74.6 (14.6)	77.6 (13.5)	0.3	
Delta	27.8 (14.5)	34.8 (14.8)	0.01*	0.48 (0.10–0.85)
I am confident in my ability to assess and manage a child with bronchiolitis.				
Before tutorial	41.8 (19.7)	39.0 (21.8)	0.5	
After tutorial	66.3 (14.6)	70.1 (15.4)	0.2	
Delta	24.5 (14.0)	31.1 (19.1)	0.03*	0.39 (0.01–0.76)
I have an improved understanding of the key history and examination findings, and differential diagnoses.	73.6 (14.4)	68.8 (16.5)	0.09	
I have an improved understanding of ordering appropriate investigations.	73.6 (13.2)	74.1 (16.9)	0.9	
I have an improved understanding of prescribing appropriate oxygen therapies.	68.0 (16.2)	73.8 (17.2)	0.07	
I have an improved understanding of how to manage fluid requirements and prescribe appropriate medications.	65.6 (17.9)	67.6 (18.3)	0.5	
I have an improved understanding of when an admission is required or when discharge is safe.	69.7 (16.9)	78.7 (16.4)	0.005*	0.54 (0.16–0.92)
I have an improved understanding of educating parents about bronchiolitis (in lay terms).	72.9 (17.0)	72.7 (18.5)	0.95	
**Gastroenteritis case responses, *n***	52	65		
I understand how to assess and manage a child with gastroenteritis and dehydration.				
Before tutorial	42.3 (16.0)	46.3 (17.2)	0.2	
After tutorial	69.9 (11.8)	73.6 (13.5)	0.1	
Delta	27.7 (11.3)	27.3 (13.7)	0.9	
I am confident in my ability to assess and manage a child with gastroenteritis and dehydration.				
Before tutorial	37.9 (16.1)	42.6 (18.0)	0.1	
After tutorial	64.1 (11.9)	70.3 (12.4)	0.007*	0.51 (0.14–0.88)
Delta	26.2 (11.6)	27.7 (15.4)	0.6	
I have an improved understanding of the key history and examination findings, and differential diagnoses.	70.7 (11.4)	70.2 (13.8)	0.8	
I have an improved understanding of assessing dehydration.	71.4 (11.7)	74.4 (15.8)	0.2	
I have an improved understanding of ordering appropriate investigations.	70.9 (10.1)	71.4 (17.0)	0.8	
I have an improved understanding of how to manage fluid requirements.	70.4 (14.4)	71.6 (17.2)	0.7	
I have an improved understanding of prescribing appropriate medications.	60.2 (17.6)	69.7 (16.6)	0.004*	0.56 (0.18–0.93)
I have an improved understanding of educating parents regarding oral rehydration at home.	59.0 (18.7)	74.1 (16.3)	<0.0001*	0.87 (0.48–1.25)

Response data from survey questions recorded using a continuous interval scale (0–100) with pre-defined categories: 1–20: Strongly Disagree; 21–40: Disagree; 41–60: Neutral; 61–80: Agree; 81–100: Strongly Agree; 0: No response. *p < 0.05.

Regarding the combined reactions from both bronchiolitis and gastroenteritis cases, PM participants reported a significantly more positive response than TT participants to the following statements: (i) ‘I enjoyed the learning activity’ [small effect size: *d* (95% CI) = 0.35 (0.08–0.61), *p* = 0.01]; (ii) ‘The learning activity was engaging’ [medium effect size: *d* (95% CI) = 0.58 (0.32–0.85), *p* < 0.0001]; (iii) ‘Receiving feedback during the learning activity aided my learning’ [medium effect size: *d* (95% CI) = 0.52 (0.26–0.78), *p* = 0.0001]; and (iv) ‘Your learning activity was more engaging than face-to-face teaching’ [large effect size: *d* (95% CI) = 0.90 (0.63–1.17), *p* < 0.0001].

A sensitivity, per-protocol analysis of the immediate reactions is presented in Supplementary Table 1.

#### 3.2.2 Immediate reactions to the educational experience: qualitative analysis (free text and open-ended questions)

##### 3.2.2.1 What was the best element of your learning activity?

**Figure 2:**
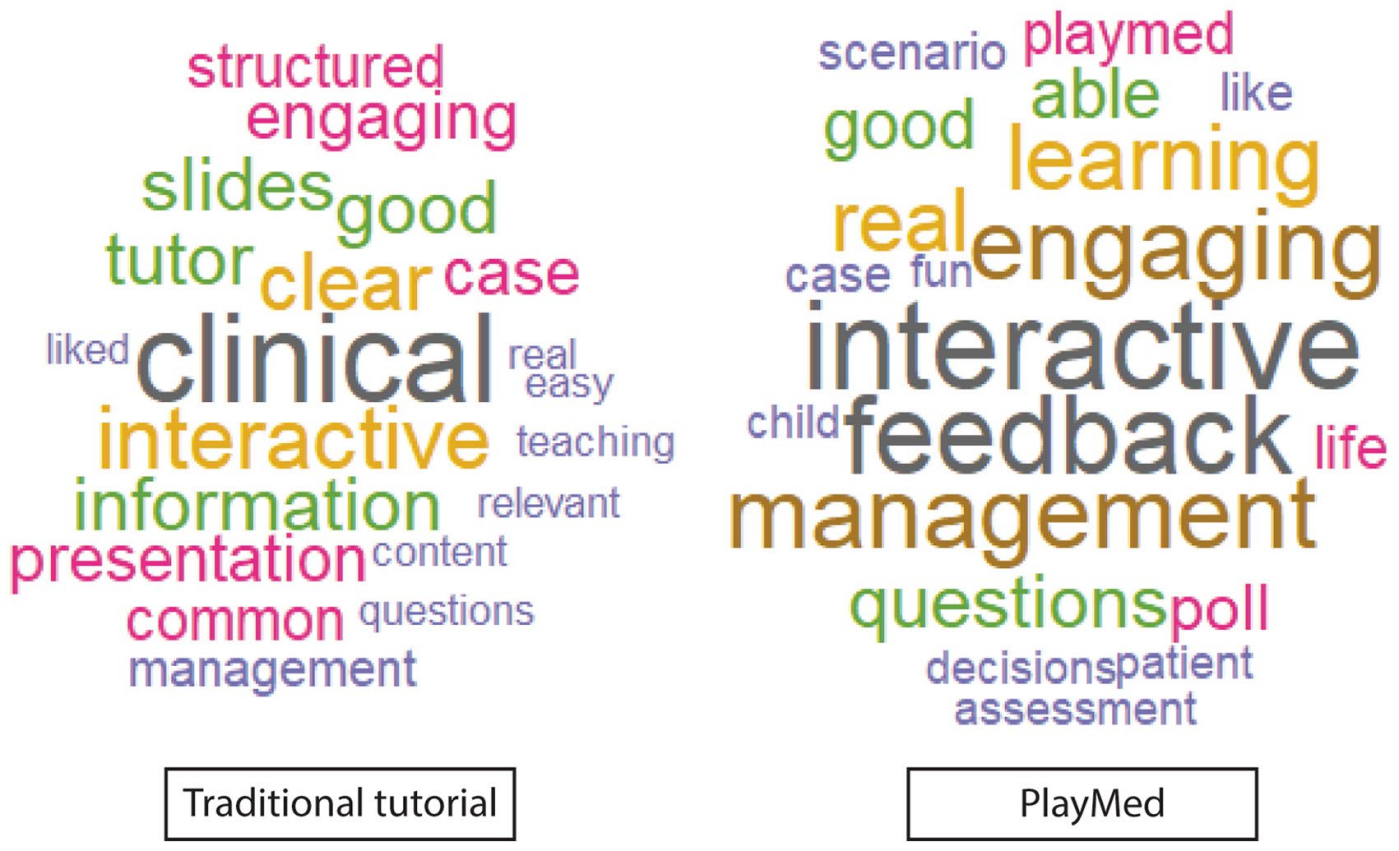
Word cloud of best elements of learning activity (immediate reaction free text).

**Figure 3:**
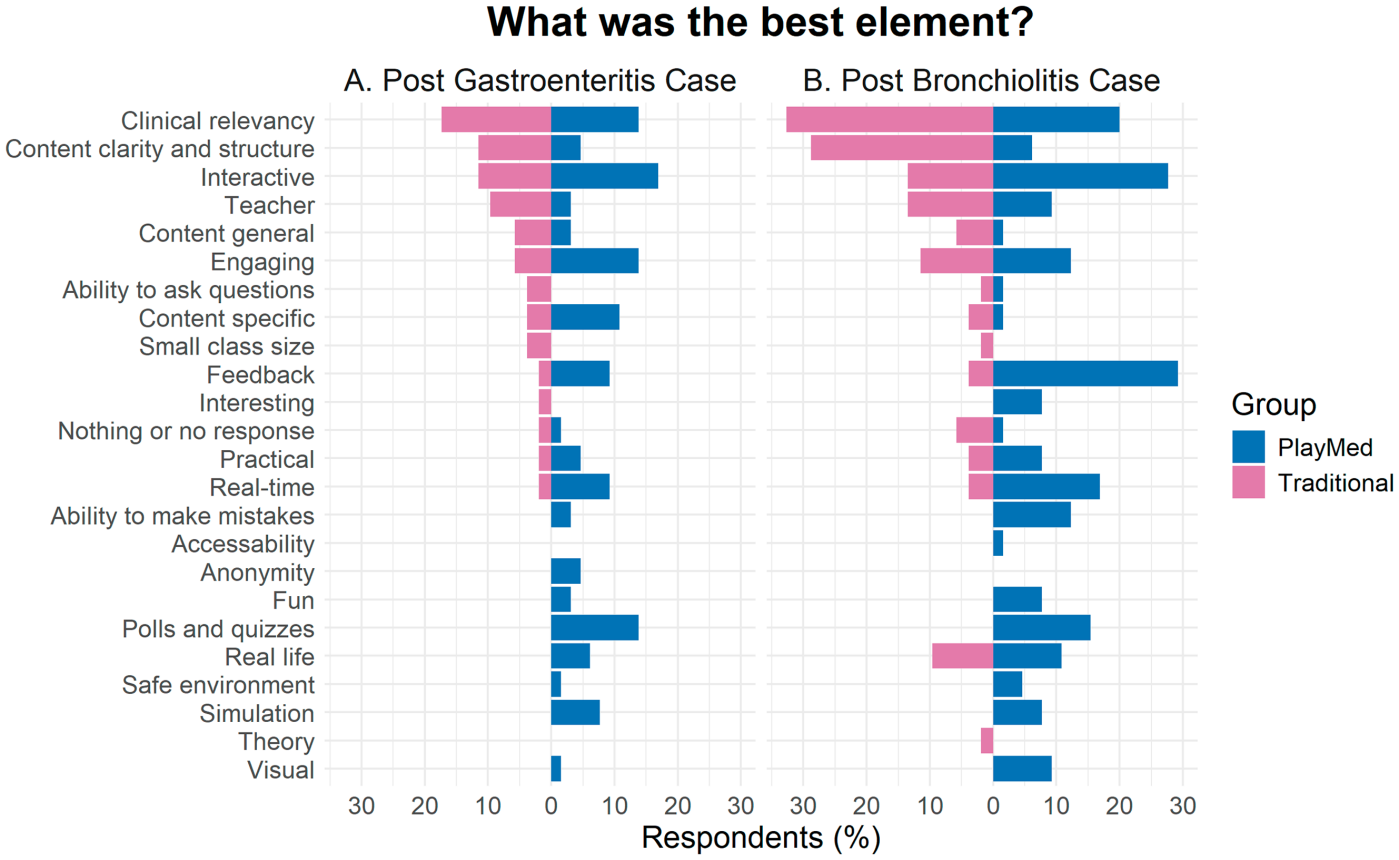
Comparison of best elements of learning post-gastroenteritis and post-bronchiolitis for PlayMed vs. Traditional tutorials (immediate reaction to open-ended questions).

Both groups described both tutorials as clinically relevant, interactive and engaging (Figure 2). In general, the TT group liked content clarity, structure and the teacher, while PM group liked that the learning activity was fun, in real time and real life. They also liked the ability to make mistakes in a safe environment with feedback (Figure 3).

##### 3.2.2.2 What could be improved?

**Figure 4:**
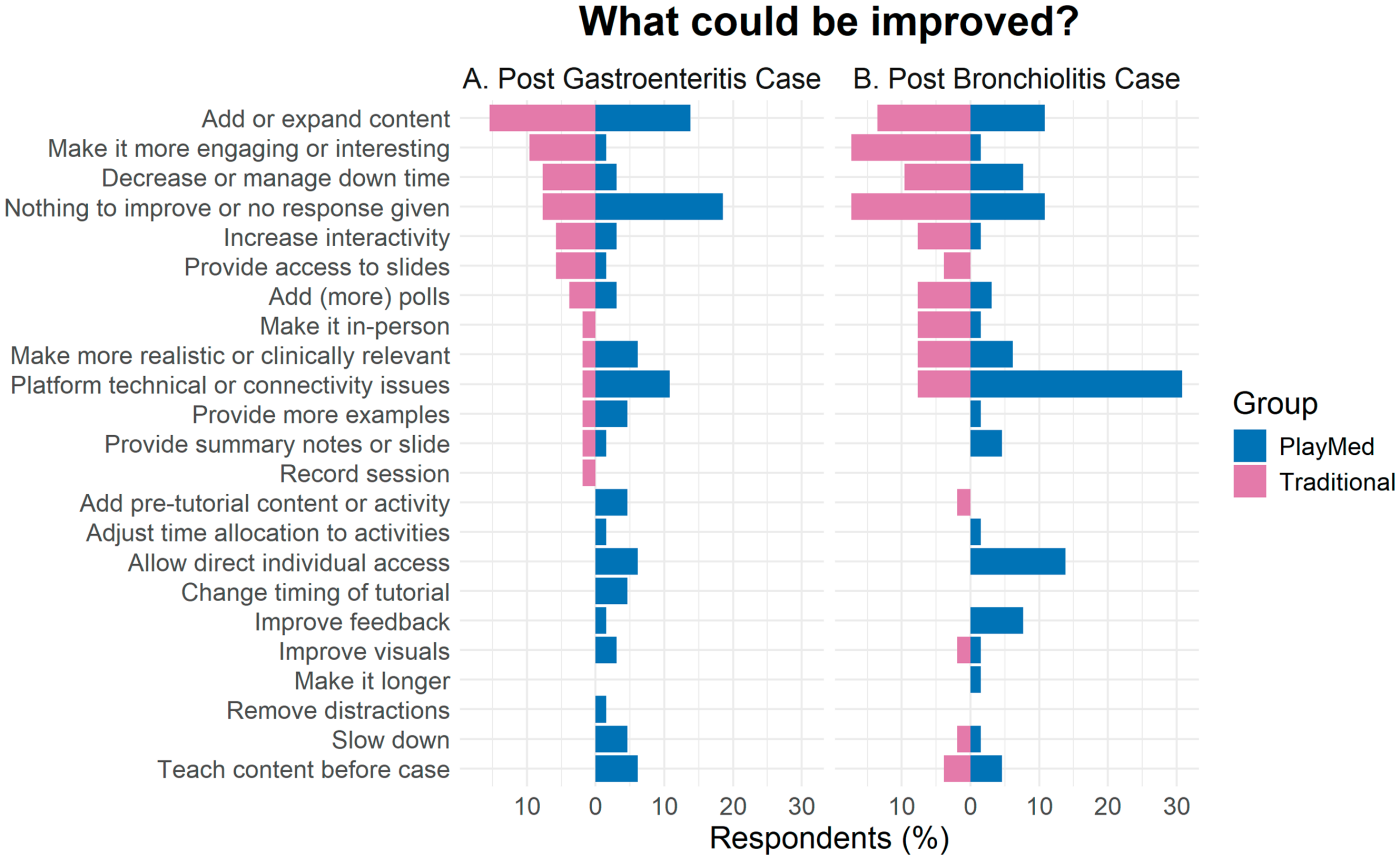
Student feedback for improvements (immediate reaction to open-ended questions).

Most students did not give a response to this or said that there was nothing to improve on (Figure 4). In both groups, add or expand content was suggested. In the PM group, majority of suggestions were to improve technology (e.g., videoconferencing glitches, connectivity issues, visuals) or allow direct individual access to the PlayMed™ software. There were also a few suggestions to have more time for the tutorials. In the TT group, suggestions included to make it more engaging or interesting, decrease or manage downtime, make the scenario more realistic or clinically relevant, and increase interactivity.

##### 3.2.2.3 What was your level of engagement with the learning activity?

**Figure 5:**
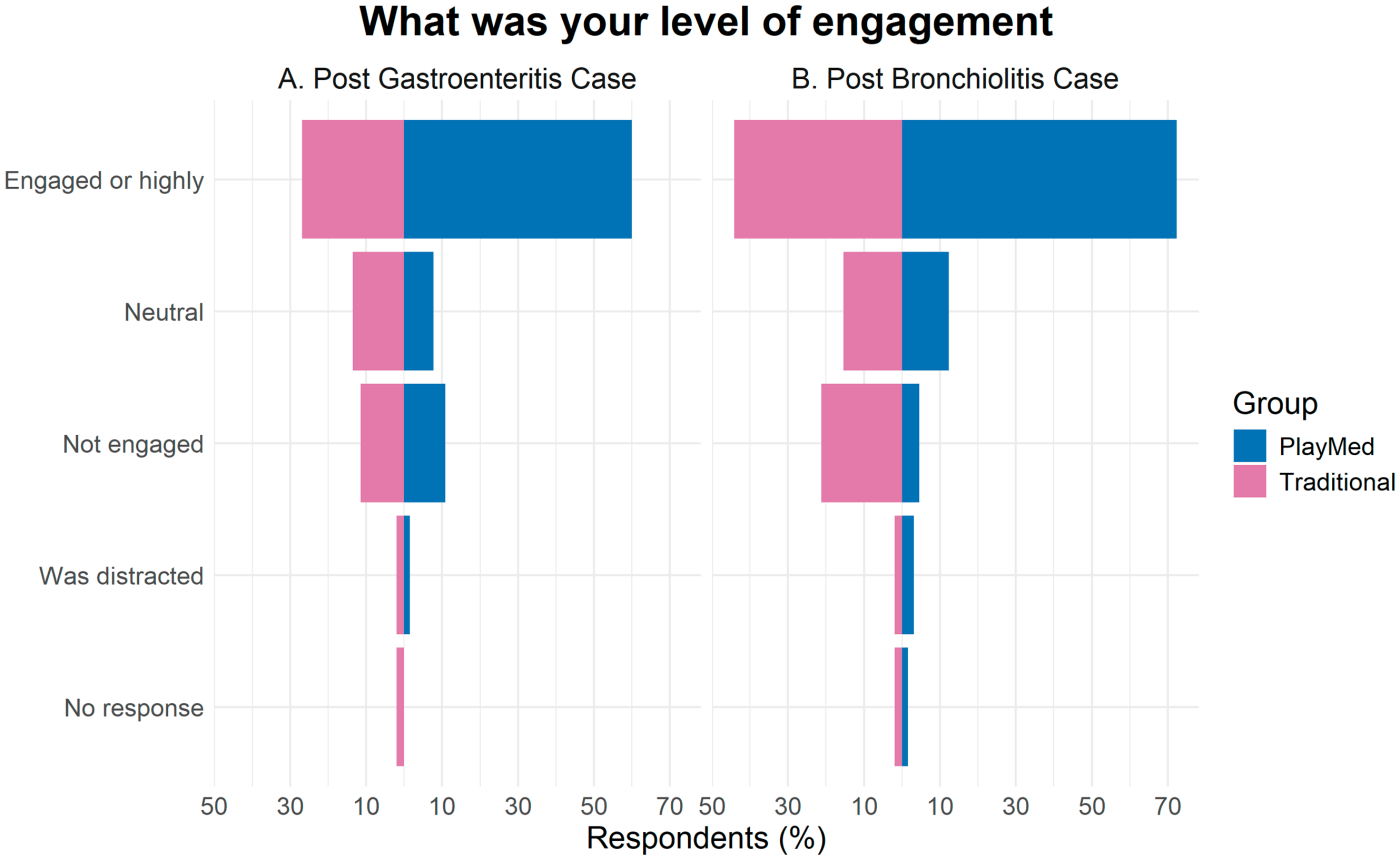
Student engagement level (immediate reaction).

Seventy-two percent and 60% of respondents from the PM group reported to be engaged or highly engaged for the bronchiolitis and gastroenteritis tutorials, respectively, (Figure 5). In comparison, 44 and 27% of the respondents from the TT group reported to be engaged or highly engaged for the respective tutorials.

##### 3.2.2.4 What was your perception of the educational value of the activity?

**Figure 6:**
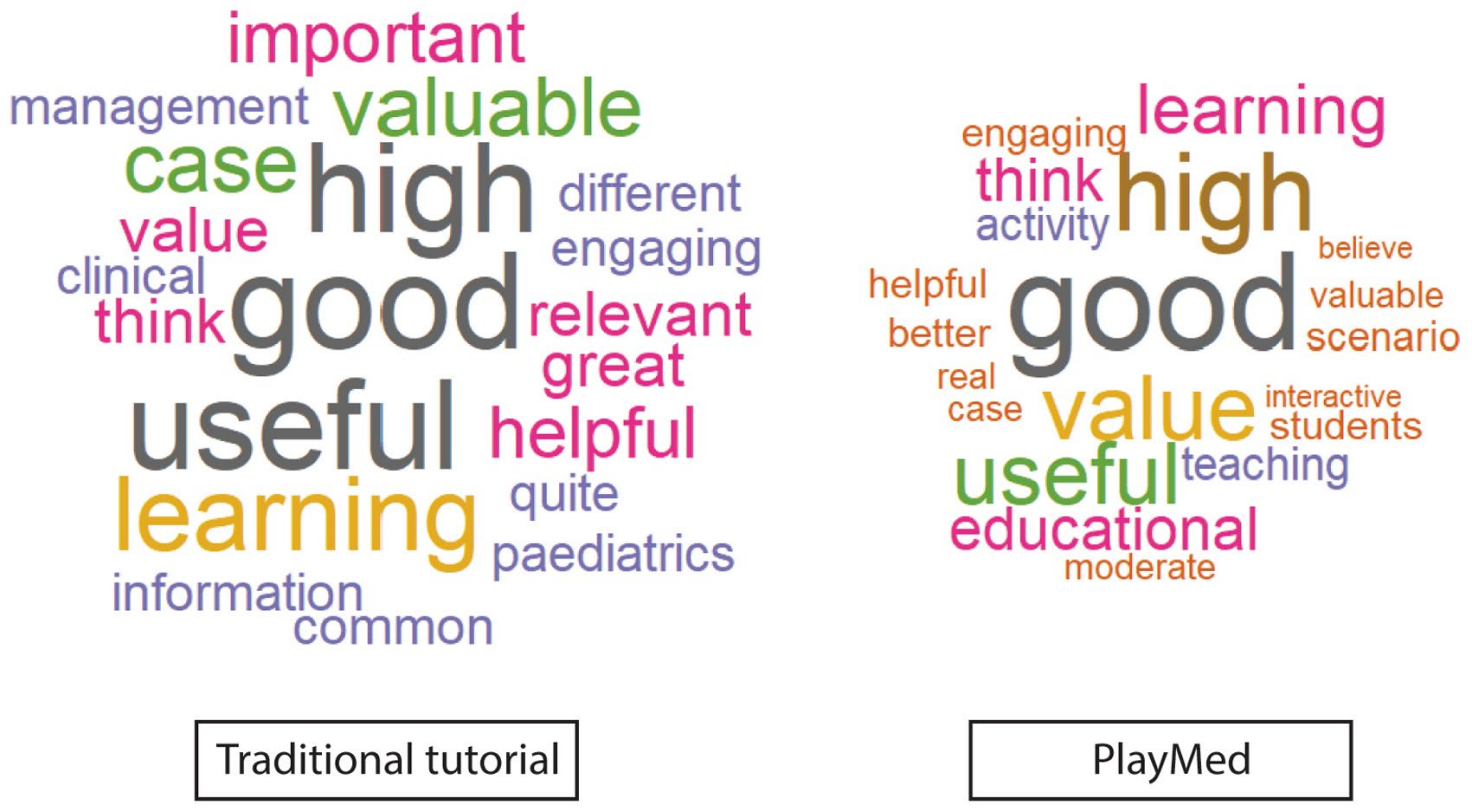
Word cloud of educational value perception (immediate reaction free texts).

Immediately post tutorials, both groups described largely positive sentiments (Figure 6).

#### 3.2.3 Teaching modality

More than three-quarters of both groups reported that the COVID-19 pandemic affected their education. When asked about what form of teaching they preferred, 24% and 21% of respondents from the TT group preferred online learning, while slightly higher proportion at 39% and 30% from the PM group did so, for the bronchiolitis and gastroenteritis tutorials, respectively. Similar proportions from both groups preferred a traditional face-to-face learning activity, at 43% (TT) and 39% (PM) respectively. Both groups wanted to do this type of online learning again, with 78% and 86% in TT group and 84 and 87% of respondents in the PM group saying yes for the bronchiolitis and gastroenteritis tutorial, respectively. Almost all the respondents (96% and 89%) in the PM group reported that they felt safe and comfortable sharing their thoughts, asking questions, or making mistakes in the gastroenteritis tutorial and bronchiolitis tutorial, respectively. A smaller proportion (76 and 74%) of the TT group reported similarly. A large majority of the students from both groups felt included in the learning activity.

### 3.3 Secondary outcome measures

The secondary aim was to evaluate and compare student learning and knowledge acquisition, assessing immediate and long-term retention of knowledge using MCQs. Additionally, the long-term reactions to the educational experience were assessed.

#### 3.3.1 Retention of knowledge—MCQs

##### 3.3.1.1 Immediately post tutorials

In a mITT analysis, the PM participants significantly outperformed TT participants on the six bronchiolitis MCQs, 4.1 (1.0) vs. 3.5 (1.0), respectively, *p* = 0.004 [medium effect size: *d* (95% CI) = 0.54 (0.16–0.91)]. There was no significant difference between PM and TT participants on the six gastroenteritis MCQs, 3.7 (1.2) vs. 3.8 (0.7), respectively, *p* = 0.7. A sensitivity, per-protocol analysis of the MCQ results, along with results for individual questions are presented in Supplementary Table 2.

##### 3.3.1.2 Week 8 post tutorials (long term)

Twenty-one participants from the PM group (32%) and eight participants from the TT group (15%) completed the week 8 MCQs. In a per-protocol analysis, there was no significant difference in the total MCQ score (out of 12) between the PM and TT groups, 8 (7–9) vs. 7.5 (6.5–8.5), respectively, *p* = 0.5. There was also no significant difference between the PM and TT groups for the six bronchiolitis MCQs (5 (4–5) vs. 4.5 (3–6), respectively, *p* = 0.98), or for the six gastroenteritis MCQs [4 (3–4) vs. 3.5 (2–4.3), *p* = 0.5]. Results for the individual questions are presented in Supplementary Table 3.

#### 3.3.2 Week 8 long term reactions to the educational experience: quantitative analysis

Eleven participants from the TT group (21%) and 25 participants from the PM group (38%) completed the week 8 long term reactions survey. A per protocol analysis was performed with responses to individual questions presented in Supplementary Table 4. Traditional tutorial participants reported significantly more positive responses than PM participants to the statement: ‘I was able to apply the acquired knowledge from my learning activity in my paediatrics term’ [medium effect size: *d* (95% CI) = 0.76 (−0.08 to 1.6), *p* = 0.01].

#### 3.3.3 Week 8 long term reactions to the educational experience: qualitative analysis (free text and open-ended questions)

##### 3.3.3.1 What was your perception of the educational value of the activity?

**Figure 7:**
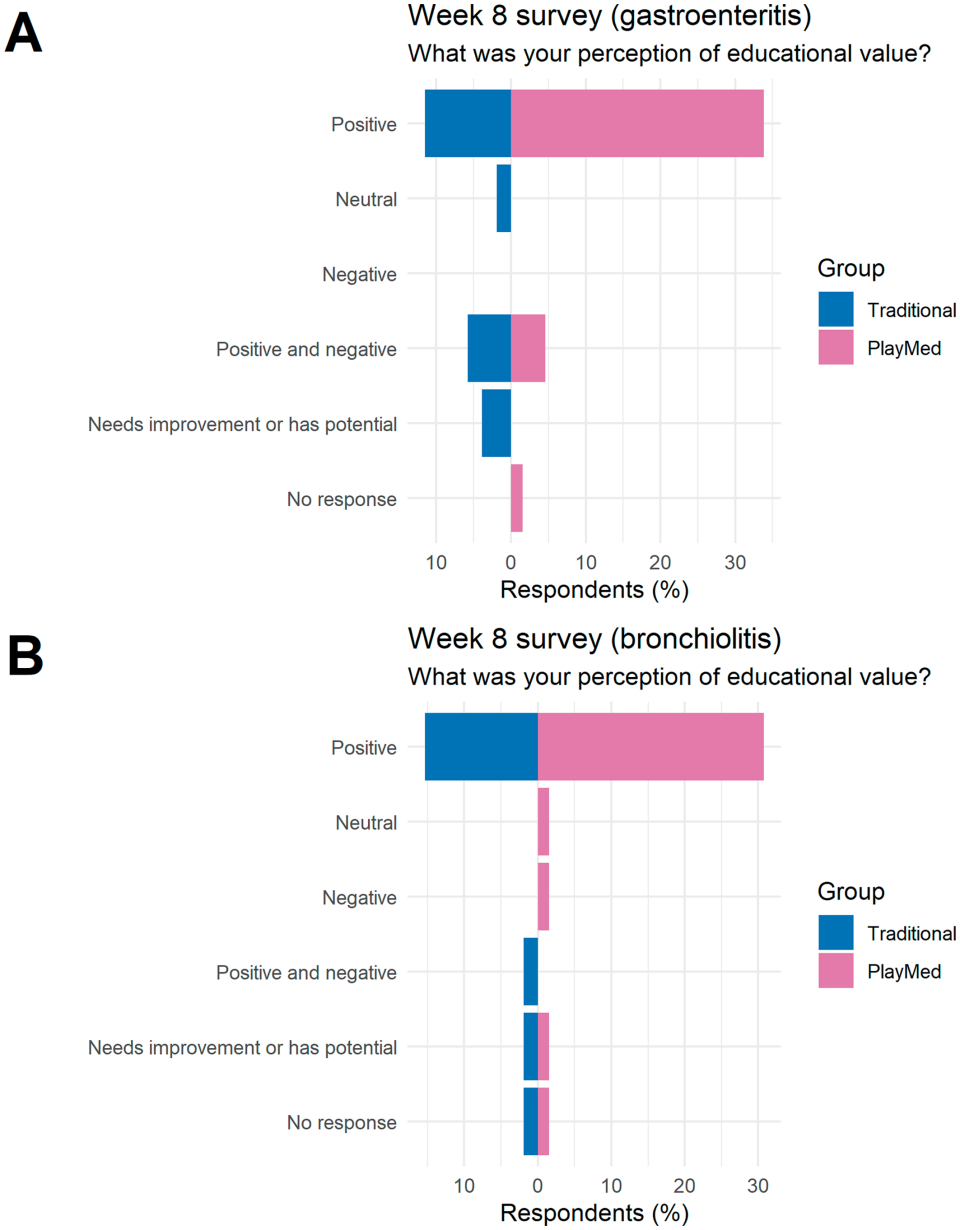
Perception of the educational value (8 weeks post-tutorial survey).

In the week 8 survey, there were more positive perceptions in the PM group vs. the TT group (Figure 7). For the gastroenteritis tutorial, 34% of respondents in the PM group responded positively compared to 12% in the TT group. For the bronchiolitis tutorial, the PM group had 31% of respondents code positively compared to 15% in the TT group.

## 4 Discussion

### 4.1 Summary of findings

By utilising a serious game (PlayMed™) to facilitate online medical student education during the COVID-19 pandemic, we demonstrated the ability to improve medical student engagement with a medium to large effect size. This mixed-methods RCT demonstrates that serious games can provide a platform to apply Kolb’s experiential learning theory (11) in a safe environment. We showed that both student reactions (Kirkpatrick Level 1) and student learning (Kirkpatrick Level 2) can be improved with utilisation of a serious game in online medical education. The utility of a serious game in medical education is likely due to the facilitation of experiential learning, which is often limited with traditional slideshow-based tutorials.

In this study, much higher levels of engagement were reported in the PM group compared to the TT group. Consistent with this, survey response rates were significantly higher in the PM group, particularly evident in the end of course surveys, reflecting higher levels of engagement in not only the short term, but also in the longer term. Higher proportions of students in the PM group reported feeling safe, compared to the TT group. These findings combined suggest that PM may relax the threat of testing. This in turn may have positive effects on knowledge acquisition as students are able to identify what they do not know and take corrective action.

Students in the PM group reported having an improved understanding of educating parents regarding oral rehydration at home in gastroenteritis (Kirkpatrick Level 1), however there was no measurable difference in both groups for the individual MCQ scores relating to this learning objective (Kirkpatrick Level 2).

For the bronchiolitis tutorial, the PM group outperformed the TT group immediately post tutorial for total MCQ scores and individual MCQ scores for two questions which assessed interpretation of clinical findings, diagnosis, and parental education about bronchiolitis (Kirkpatrick Level 2).

### 4.2 Comparison with literature

There is a growing body of evidence that using serious games in healthcare professions education can increase engagement, fuel motivation to learn and improve learning outcomes (24). However, there has been little high-quality translational research in this context, especially using a randomised controlled trial to evaluate and compare learner attitudes and knowledge (i.e., Kirkpatrick’s Model of Evaluation Levels 1 and 2) (25). To our knowledge, this is the first randomised controlled trial that has explored using serious games in a case-based group tutorial setting in medical education. In most of the literature, serious games were used in individual player or multiplayer settings where the learner interacts with the game and other learners, without a tutor present (26–31). Integrating PM in the delivery of tutorials uniquely involves live facilitation by the tutor, enabling tutor assessment of student group competency levels, matching of students with appropriate task complexity. This may possibly mitigate the ‘expertise reversal effect’ (32), while still encouraging development of collaborative awareness, clinical reasoning and decision-making skills (9, 33, 34). Feedback in the PM tutorial is delivered real-time, dually by the game programme and the tutor, which aims to achieve timely, specific, and actionable feedback, fundamental for learners’ clinical skill development (35).

In our study, possible reasons for higher engagement levels in the PM group include adding a fun dimension to the learning activity, real time case evolution dependent on the group’s clinical management decisions and simulating real life patient scenarios. Polls and quizzes integrated into the teaching session helped structure the exploration of clinical decision making.

A large majority of students in the PM group reported positively on psychological safety, affirming that this is a safe educational intervention. Psychological safety is defined as a shared belief that the team is safe for interpersonal risk taking (36). When fostered in education, students have improved engagement, participation and learning experience (37, 38). Factors contributing to psychological safety in the PM tutorials include anonymity in polls and quizzes, which remove risk of exposure or judgement. Students exercised their personal choice to keep their webcams on or off; reinforcing social presence when on but reducing the risk of judgement when webcams were off.

Students in the PM group had higher MCQ scores compared to the TT group for the bronchiolitis tutorial, suggesting that the PM group had improved knowledge acquisition and clinical reasoning, which includes interpretation of symptoms, examination findings, formulating diagnoses and initiating appropriate management (39). Similarly, previous trials have demonstrated positive implications of serious games on teaching knowledge (31, 40). Interestingly, the PM group did not show superior MCQ scores for the gastroenteritis tutorial, raising the possibility that some of the benefits conferred when using a serious game like PM may be case-specific. Unfortunately, there is no strong evidence on the role of case specificity in transferability of clinical reasoning (41) and literature is scarce on which components of clinical reasoning are transferable.

### 4.3 Limitations

This was a single-centre study with limited sample size recruiting students in their final 2 years of medical school, relating to paediatric-specific content, which limits generalizability of results and external validity. Drop-out rate from attendance at tutorials to combined survey completion (immediate post tutorial and week 8 surveys) were higher in the TT group at 29% versus 19% in the PM group. This may reflect higher participant engagement in the PM group rather than a random effect. We measured outcomes in the short to long term (end of 8-week course) but were not able to measure real-world clinical effects resembling the third and fourth levels of the Kirkpatrick Model.

Execution of the PM tutorial was not always consistently smooth, due to limitations in connectivity and high-resolution visuals. Despite this, large majority of the PM group reported wanting to do this type of online learning again. Given that the students in both groups preferred traditional face-to-face teaching, using PM in a face-to-face setting may bypass these technology challenges and boost educational experience.

## 5 Conclusion

This mixed-methods RCT provides support for the development and utilisation of serious games for online medical student education. We demonstrated that a novel serious game such as PlayMed™, can provide a superior educational experience compared to traditional slideshow-based presentations. Furthermore, a serious game can provide a safe learning environment, facilitating students’ experiential learning. Further research assessing participants’ real-world clinical behaviour in a larger multicentre trial is required.

## Data Availability

The original contributions presented in the study are included in the article/Supplementary material, further inquiries can be directed to the corresponding author.

## Footnotes

^1^http://www.sealedenvelope.com
