# Supplementary material for: Serious games vs. traditional tutorials in the pandemic: a randomised controlled trial
Source: Front Med (Lausanne). 2024 Nov 21;11:1424024. doi: 10.3389/fmed.2024.1424024 (PMC11617181; doi:10.3389/fmed.2024.1424024)
Supplement: Supplementary file 1 [file Data_Sheet_1.PDF]

Supplementary Material

Serious games versus traditional tutorials in the pandemic: a randomised controlled trial

Su Min Joyce Tan, Michael J. Coffey, Katrina Blazek, Neela Sitaram, Isabella Dobrescu, Alberto Motta, Sandra Chuang, Chee Y. Ooi*

*** Correspondence:** A/Prof (Keith) Chee Y. Ooi keith.ooi@unsw.edu.au

1 Appendix 1A: Immediate feedback survey post tutorial on educational experience

1.1 Statement ratings

Please indicate to what extent you agree or disagree with the following statements about the learning activity provided to you. Please respond on the fixed interval sliding scale (0 to 100) which uses the following definitions:

0: No response
1 – 20: Strongly Disagree
21 – 40: Disagree
41 – 60: Neutral
61 – 80: Agree
81 – 100: Strongly Agree

- The learning activity will help me prepare for and deal with real-life clinical scenarios.
- I enjoyed the learning activity.
- The learning activity was engaging.
- I was able to understand the content delivered during the learning activity.
- Receiving feedback during the learning activity aided my learning.
- I believe this learning activity will be beneficial for my paediatrics term.
- I would attend a similar learning activity in the future.
- I would recommend this learning activity to a colleague.
- The learning activity was more engaging than face-to face teaching.

1.1.1 Specific to gastroenteritis tutorial only

- I understand how to assess and manage a child with gastroenteritis and dehydration.
- I am confident in my ability to assess and manage a child with gastroenteritis and dehydration.
- I have an improved understanding of the key history and examination findings, and differential diagnoses.
- I have an improved understanding of assessing dehydration.
- I have an improved understanding of how to manage fluid requirements.

- I have an improved understanding of prescribing appropriate medications.
- I have an improved understanding of educating parents regarding oral rehydration at home.

1.1.2 Specific to bronchiolitis tutorial only

- I understand how to assess and manage a child with bronchiolitis.
- I am confident in my ability to assess and manage a child with bronchiolitis.
- I have an improved understanding of the key history and examination findings, and differential diagnoses.
- I have an improved understanding of ordering appropriate investigations.
- I have an improved understanding of how to manage fluid requirements and prescribe appropriate medications.
- I have an improved understanding of when an admission is required or when discharge is safe.
- I have an improved understanding of educating parents about bronchiolitis (in lay terms).

1.2 Free text or open-ended questions

- What was the best element of your learning activity?
- What could be improved?
- What was your level of engagement with the learning activity?
- What is your perception of the educational value of the learning activity?

1.3 Multiple choice questions with free text option

- What form of teaching do you prefer? Choose one of the below:
  - Traditional face-to-face learning
  - Online learning activity
  - I am indifferent between the two approaches above
  - Please explain why.
- Would you want to do this type of online learning again?
  - Yes
  - No
  - Please explain why.
- Did you feel safe and comfortable sharing your thoughts, asking questions or making mistakes?
  - Yes
  - No
  - Please explain why.
- Did you feel included in the learning activity?
  - Yes
  - No
  - Please explain why.
- Do you feel the COVID-19 pandemic affected your education?
  - Yes
  - No

- Please explain why.

2 Appendix 1B: End of course feedback survey on educational experience

2.1 Statement ratings

2.1.1 Specific to gastroenteritis tutorial only

Please indicate to what extent you agree or disagree with the following statements about the learning activity provided to you. Please respond on the fixed interval sliding scale (0 to 100) which uses the following definitions:

0: No response
1 – 20: Strongly Disagree
21 – 40: Disagree
41 – 60: Neutral
61 – 80: Agree
81 – 100: Strongly Agree

- The learning activity was beneficial for my paediatrics term.
- The learning activity motivated me to learn more about gastroenteritis and dehydration.
- I was satisfied with the method in which my learning activity was conducted.
- I understand how to assess and manage a child with gastroenteritis and dehydration.
- I am confident in my ability to assess and manage a child with gastroenteritis and dehydration.
- The learning activity helped me prepare for, understand and deal with real-life clinical scenarios.
- The learning activity impacted on my paediatrics term.
- I am more confident in my ability to assess and manage gastroenteritis and dehydration because of my learning activities.

In a child with gastroenteritis:

- I have an improved understanding of the key history and examination findings, and differential diagnoses.
- I have an improved understanding of assessing dehydration.
- I have an improved understanding of ordering appropriate investigations.
- I have an improved understanding of how to manage fluid requirements.
- I have an improved understanding of prescribing appropriate medications.
- I have an improved understanding of educating parents regarding oral rehydration at home.

2.1.2 Specific to bronchiolitis tutorial only

Please indicate to what extent you agree or disagree with the following statements about the learning activity provided to you. Please respond on the fixed interval sliding scale (0 to 100) which uses the following definitions:

0: No response
1 – 20: Strongly Disagree
21 – 40: Disagree
41 – 60: Neutral
61 – 80: Agree
81 – 100: Strongly Agree

- The learning activity was beneficial for my paediatrics term.
- The learning activity motivated me to learn more about bronchiolitis.
- I was satisfied with the method in which my learning activity was conducted.
- I understand how to assess and manage a child with bronchiolitis.
- I am confident in my ability to assess and manage a child with bronchiolitis.
- The learning activity helped me prepare for, understand and deal with real-life clinical scenarios.
- The learning activity impacted on my paediatrics term.
- I am more confident in my ability to assess and manage bronchiolitis because of my learning activities.

In a child with bronchiolitis:

- I have an improved understanding of the key history and examination findings, and differential diagnoses.
- I have an improved understanding of ordering appropriate investigations.
- I have an improved understanding of prescribing appropriate oxygen therapies.
- I have an improved understanding of how to manage fluid requirements and prescribe appropriate medications.
- I have an improved understanding of when an admission is required or when discharge is safe.
- I have an improved understanding of educating parents about bronchiolitis (in lay terms).

2.2 Free text or open-ended questions

- What is your perception of the educational value of the learning activity?
- I was able to apply the acquired knowledge from my learning activity in my paediatrics term.
    - If so, how/provide an example.

3 Appendix 2A: Multiple choice questions for Gastroenteritis

Q1. Which observation on his bedside chart indicates a severe degree of dehydration?

A. Capillary refill of 3 seconds
B. A heart rate of 120 beats per minute
C. A respiratory rate of 50 breaths per minute
D. A blood pressure of 70/50mmHg
E. A temperature of 38° C

Answer: D

Q2. An 18 month old boy with acute gastroenteritis and mild dehydration presents to the emergency department. They have had two days of non-bilious vomiting and abdominal pain. One day of watery diarrhoea without blood or mucus. Fevers to 38 degrees today. No sick contacts in the immediate family. No recent travel history. They attend daycare two days per week. Their weight is 10kg. Which of the following medications AND indications is MOST appropriate:

A. Loperamide to reduce the amount of diarrhoea
B. Metoclopramide to stop vomiting and facilitate discharge home
C. Metronidazole for empiric cover of bacterial gastroenteritis
D. Ondansetron to stop vomiting and enable a trial of oral fluid
E. Probiotics to restore normal gut flora

Answer: D

Q3. Which presentation is the most consistent with viral gastroenteritis?

A. A 6 week old infant with a 3 day history of projectile, non-bilious vomiting and reduced urine output
B. A 3 year old boy with a 24 hour history of cough, vomiting, loose stools and a blanching rash
C. A 6 month girl with a 24 hour history of fever, vomiting and malodorous urine
D. A 3 year old boy with a 4 week history of vomiting, malaise and 3kg of weight loss
E. A 6 year old girl with a 48 hour history of fever, vomiting and abdominal pain including percussion tenderness in the right lower quadrant

Answer: B

Q4. A 3 year old girl presents with three day history of 2-3 loose stools a day with vomiting over the last 12 hours. The past few days she has been drinking a bit more than half her usual intake but in the last 24 hours, she's had 6 vomits. Her parents say that she's probably kept down 200ml of juice. She also ate a few pieces of fruit but vomited all the food up. She's been more tired than usual and not her usual self and complaining of occasional tummy pain. On examination she has RR 35, HR 140, oxygen saturation 98% room air, Temperature 37.5C. Her abdomen is soft and non-tender on examination. She has dry lips.

What investigations and management would you consider for this patient?

A. Perform urinalysis and give aggressive oral hydration
B. Check electrolytes, perform a stool culture and urinalysis, start IV fluids at maintenance
C. Give a 10ml/kg fluid bolus and start fluids at maintenance +5%.
D. Perform stool culture, give loperamide and trial oral rehydration
E. Check blood glucose level and start NG or IV rapid rehydration

Answer: E

Q5. A 8 year old boy presents with gastroenteritis and who appears moderately dehydrated with ongoing large amounts of watery, non-bloody diarrhoea. You have assessed him and

decided to give him IV maintenance plus 3% for replacement. He weighs 28kg. What type and what rate of fluid should he be given?

A. 0.9% sodium chloride, at 69ml/hr
B. 0.9% sodium chloride + 5% dextrose at 69ml/hr
C. 0.9% sodium chloride + 5% dextrose at 75ml/hr
D. 0.9% sodium chloride + 5% dextrose at 104ml/hr
E. 0.9% sodium chloride +5 % dextrose at 127ml/hr

Answer: D

Q6. With regards to oral rehydration/feeding at home, which of the following pieces of advice should NOT be given to parents?

A. Small frequent amounts of fluid should be offered. 5mL/kg/hr is a good amount to aim for while your child is awake.
B. Oral rehydration solutions (ORS) bought from a chemist are specially formulated to replace lost fluid and take advantage of the sodium-glucose co-transporter in the small intestine
C. Clear fluids such as juice or cordial can be used as alternative to ORS, but should be diluted to avoid worsening diarrhoea due to higher carbohydrate content
D. Breastfed infants should stop breastfeeding completely for 24-48 hours in favour of premade oral rehydration solution
E. It is safe to re-introduce age appropriate foods at mealtimes as soon as the child is interested even if diarrhoea is ongoing

Answer: D

4 Appendix 2B: Multiple choice questions for Bronchiolitis

Case stem for Q1 and Q2: *A 5 month old female infant presents with her mother with difficulty breathing overnight. She has had rhinorrhoea and cough for 3 days and a low grade temperature to 37.8 degrees. She is an otherwise well child with no past medical history of note. She has an older brother who is 3 years of age who attends daycare and also had viral upper respiratory tract infection. He has previously required admission to hospital with respiratory illnesses needing salbutamol and oxygen. Her mother reports that there is a family history of asthma and hayfever. She is exclusively breastfed and feeding well with normal wet nappies.*

On examination, she is alert and active, warm and well perfused with a capillary refill time of 2 seconds. Her heart rate is 130 bpm and respiratory rate is 55/minute. She has intercostal and subcostal recession. On auscultation of her chest, she has diffuse wheeze and scattered crepitations and air entry is normal and equal bilaterally. Her oxygen saturation is 97% on room air.

Q1. What is the most likely diagnosis?

A. Bacterial or atypical pneumonia
B. Croup
C. Viral Induced Wheeze /Asthma

D. Viral bronchiolitis
E. Upper Respiratory Tract Infection

Answer: D

Q2. What further features on history or examination is LEAST likely to indicate that admission is warranted?

A. Grunting or nasal flare
B. Oxygen levels drop with feeding to 87% but she is well in herself and feeding well
C. The family lives far away and the parents are anxious
D. She develops a temperature to 38.5 degrees
E. Her blood glucose level is 2.0mmol/L

Answer: D

Case stem for Q3 and Q4: *Freddie is a 5 month old boy who is brought to the emergency department by his mother as she is concerned that he looks like he is struggling to breathe. 2 days ago he developed a runny nose, then yesterday he started coughing. Today his breathing seems much faster and he is breast feeding for half the usual amount of time before tiring and falling asleep. His nappy has been dry for the past 8 hours. Freddie was born at 40 weeks gestation, has no known medical issues and has received immunisations as per the recommended Australian Childhood Immunisation schedule.*

On examination, his vital signs are: RR 84, O2 86% RA, HR 170, BP 90/50, T 38.4. He appears lethargic and mottled with severe increased work of breathing (head bobbing, suprasternal, intercostal and subcostal recession). He feels cool peripherally with central capillary refill time of 5 seconds. He has normal 1st and 2nd heart sounds with no murmur. He has widespread wheeze and scattered crepitations with equal air entry bilaterally.

A provisional diagnosis of bronchiolitis is made.

Q3. Which of the following statements is incorrect about bronchiolitis?

A. Risk factors for more severe disease include prematurity, congenital heart disease and immunodeficiency
B. Nasopharyngeal aspirates to test for common respiratory viruses are important to confirm the diagnosis
C. Bronchiolitis is the most likely diagnosis for an infant who presents to the emergency department with increased work of breathing.
D. Typically, infants with bronchiolitis have a peak of symptom severity on day 3-5, and may continue to cough for up to 2 weeks after onset of symptoms
E. Chest X-rays are generally considered to be unnecessary in confirming the diagnosis

Answer: B

Q4. Which of these statements is true about the management of bronchiolitis?

A. Nasogastric fluid replacement is preferable to the intravenous route in children admitted to hospital with mild disease
B. Providing warm and humidified air can reduce severity of symptoms at home
C. Antibiotics should be routinely prescribed for all children with bronchiolitis
D. Salbutamol has an important role as adjunctive therapy
E. Corticosteroids, such as prednisolone have been shown in studies to decrease length of hospital stay

Answer: A

Q5. A 5-month-old with bronchiolitis presents to the emergency department with moderate to severe increased work of breathing, oxygen saturations of 86-88% in room air, a respiratory rate of 80 breaths per minute and weighs 7kg. Which of the following management options is most appropriate?

A. Sit upright on parent's lap
B. Low-flow nasal prongs (LFNP) at 2L/min
C. High-flow nasal prongs (HFNP) at 14L/min
D. Continue positive airway pressure (CPAP) at 8cmH20
E. Bilevel positive airway pressure (BiPAP) with inspiratory pressure of 13cmH20 and expiratory pressure of 7cmH20

Answer: C

Q6. When educating parents about bronchiolitis, which of the following pieces of advice should NOT be given?

A. Children may have transient increased work of breathing after feeding
B. An episode bronchiolitis in infancy is a sign your child is likely to develop asthma in later life
C. The pathophysiology of bronchiolitis is different to asthma and therefore the use of bronchodilators such as salbutamol are not recommended as they have not been shown to improve outcomes
D. Smaller volume, increased frequency oral feeds should be offered
E. Persistently worse work of breathing is a sign you should return to hospital for review

Answer: B

5 Supplementary Table 1: Immediate reactions to the educational experience

	Intention-to-treat Analysis				Per Protocol Analysis			
	**Traditional Tutorial**	**PlayMed Tutorial**	**p-value**	***d* (95% CI)**	**Traditional Tutorial**	**PlayMed Tutorial**	**p-value**	***d* (95% CI)**
**Number of participants, n.**	52	65			50	64		
**Included survey responses**								
Post bronchiolitis case, n.	52	65			44	58		
Post gastroenteritis case, n.	52	65			28	54		
Total, n	104	130			72	112		
**Reactions (post bronchiolitis & gastroenteritis cases)**								
I enjoyed the learning activity.	69.6 (18.8)	75.7 (16.5)	0.01*	0.35 (0.08 – 0.61) small	69.8 (22.6)	75.8 (17.8)	0.06	
The learning activity was engaging.	68.7 (19.4)	78.9 (16.0)	<0.0001*	0.58 (0.32 – 0.85) medium	69.2 (23.3)	79 (17.2)	0.003*	0.496 (0.19 – 0.80) small
I was able to understand the content delivered during the learning activity.	82.9 (14.4)	79.7 (15.6)	0.1		83.9 (17.0)	79.8 (16.7)	0.1	
I believe this learning activity will be beneficial for my paediatrics term.	79.2 (15.8)	80.4 (14.7)	0.6		79.6 (18.9)	80.4 (15.9)	0.8	
The learning activity will help me prepare for and deal with real-life clinical scenarios.	74.9 (14.9)	75.2 (15.0)	0.9		74.8 (17.9)	75.2 (16.1)	0.9	

I would attend a similar learning activity in the future.	79.8 (17.0)	79.0 (18.7)	0.7		79.9 (20.5)	79.0 (20.2)	0.8	
I would recommend this learning activity to a colleague.	74.5 (18.7)	77.3 (19.3)	0.1		73.6 (22.5)	77.3 (20.9)	0.3	
Receiving feedback during the learning activity aided my learning.	67.1 (21.0)	77.4 (18.5)	0.0001*	0.52 (0.26 – 0.78) medium	67.4 (25.3)	77.4 (20.0)	0.006*	0.45 (0.15 – 0.75) small
Your learning activity was more engaging than face-to-face teaching.	43.6 (21.0)	63.9 (23.8)	<0.0001*	0.90 (0.63 – 1.17) large	36.0 (29.8)	56.6 (32.8)	<0.0001*	0.75 (0.42 – 1.08) medium
**Bronchiolitis case specific reactions**								
I understand how to assess and manage a child with bronchiolitis.								
Before tutorial	46.8 (19.1)	42.8 (19.6)	0.3		46.8 (21.3)	42.8 (20.8)	0.4	
After tutorial	74.6 (14.6)	77.6 (13.5)	0.3		74.6 (16.3)	77.6 (14.4)	0.3	
Delta	27.8 (14.5)	34.8 (14.8)	0.01*	0.48 (0.10 – 0.85) small	27.8 (16.2)	34.8 (15.7)	0.03*	0.44 (0.03 – 0.85) small
I am confident in my ability to assess and manage a child with bronchiolitis.								
Before tutorial	41.8 (19.7)	39.0 (21.8)	0.5		41.8 (22.0)	39.0 (23.1)	0.5	
After tutorial	66.3 (14.6)	70.1 (15.4)	0.2		66.3 (16.3)	70.1 (16.4)	0.3	
Delta	24.5 (14.0)	31.1 (19.1)	0.03*	0.39 (0.01 – 0.76)	24.5 (15.6)	20.3 (31.1)	0.07	

				small				
I have an improved understanding of the key history and examination findings, and differential diagnoses.	73.6 (14.4)	68.8 (16.5)	0.09		73.6 (16.1)	68.8 (17.5)	0.2	
I have an improved understanding of ordering appropriate investigations.	73.6 (13.2)	74.1 (16.9)	0.9		73.6 (14.7)	74.1 (17.9)	0.9	
I have an improved understanding of prescribing appropriate oxygen therapies.	68.0 (16.2)	73.8 (17.2)	0.07		68.0 (18.1)	73.8 (18.2)	0.12	
I have an improved understanding of how to manage fluid requirements and prescribe appropriate medications.	65.6 (17.9)	67.6 (18.3)	0.5		65.6 (20.0)	67.6 (19.4)	0.6	
I have an improved understanding of when an admission is required or when discharge is safe.	69.7 (16.9)	78.7 (16.4)	0.005*	0.54 (0.16 – 0.92) medium	69.7 (18.8)	78.7 (17.4)	0.02*	0.498 (0.09 – 0.91) small
I have an improved understanding of educating parents about bronchiolitis (in lay terms).	72.9 (17.0)	72.7 (18.5)	0.95		72.9 (18.9)	72.7 (19.7)	0.96	
**Gastroenteritis case specific reactions**								
I understand how to assess and manage a child with gastroenteritis and dehydration.								
Before tutorial	42.3 (16.0)	46.3 (17.2)	0.2		42.3 (22.0)	46.3 (19.1)	0.4	
After tutorial	69.9 (11.8)	73.6 (13.5)	0.1		70.0 (16.2)	73.6 (15.0)	0.3	
Delta	27.7 (11.3)	27.3 (13.7)	0.9		27.7 (15.5)	27.3 (15.2)	0.9	
I am confident in my ability to assess and manage a child with gastroenteritis and dehydration.								
Before tutorial	37.9 (16.1)	42.6 (18.0)	0.1		37.9 (22.1)	42.6 (20.0)	0.3	

After tutorial	64.1 (11.9)	70.3 (12.4)	0.007*	0.51 (0.14 – 0.88)	64.1 (16.2)	70.3 (13.7)	0.09	
Delta	26.2 (11.6)	27.7 (15.4)	0.6		26.2 (15.9)	27.7 (17.1)	0.7	
I have an improved understanding of the key history and examination findings, and differential diagnoses.	70.7 (11.4)	70.2 (13.8)	0.8		70.7 (15.7)	70.2 (15.4)	0.9	
I have an improved understanding of assessing dehydration.	71.4 (11.7)	74.4 (15.8)	0.2		71.4 (16.0)	74.4 (17.5)	0.4	
I have an improved understanding of ordering appropriate investigations.	70.9 (10.1)	71.4 (17.0)	0.8		70.9 (13.9)	71.4 (18.9)	0.9	
I have an improved understanding of how to manage fluid requirements.	70.4 (14.4)	71.6 (17.2)	0.7		70.4 (19.8)	71.6 (19.1)	0.8	
I have an improved understanding of prescribing appropriate medications.	60.2 (17.6)	69.7 (16.6)	0.004*	0.56 (0.18 – 0.93) medium	60.2 (24.2)	69.7 (18.4)	0.08	
I have an improved understanding of educating parents regarding oral rehydration at home.	59.0 (18.7)	74.1 (16.3)	<0.0001*	0.87 (0.48 – 1.25) large	59.0 (25.7)	74.1 (18.0)	0.009*	0.72 (0.24 – 1.20) large

Response data from survey questions recorded using a continuous interval scale (0 to 100) with pre-defined categories: 1 - 20: Strongly Disagree; 21 - 40: Disagree; 41 - 60: Neutral; 61 - 80: Agree; 81 - 100: Strongly Agree; 0: No response. * p<0.05.

6 Supplementary Table 2: Immediate retention of knowledge – MCQ results

	**Intention-to-treat Analysis**				**Per Protocol Analysis**			
	**Traditional Tutorial**	**PlayMed Tutorial**	**p-value**	***d* (95% CI)**	**Traditional Tutorial**	**PlayMed Tutorial**	**p-value**	***d* (95% CI)**
**Number of participants, n.**	52	65			50	64		
**Immediate MCQ responses**								
Post bronchiolitis case, n.	52	65			37	58		
Mean score out of 6	3.5 (1.0)	4.1 (1.0)	0.004*	0.54 (0.16 – 0.91) Medium	3.5 (1.1)	4.1 (1.1)	0.03*	0.48 (0.06 – 0.91) small
What is the most likely diagnosis?					73%	90%	0.07	
What further features on history or examination is LEAST likely to indicate that admission is warranted?					8%	22%	0.1	
Which of the following statements is incorrect about bronchiolitis?					92%	90%	0.99	
Which of these statements is true about the management of bronchiolitis?					59%	55%	0.8	
Which of the following management options is most appropriate?					41%	52%	0.4	
When educating parents about bronchiolitis, which of the following pieces of advice should NOT be given?					81%	98%	0.01	
Post gastroenteritis case, n.	52	65			26	51		
Mean score out of 6	3.8 (0.7)	3.7 (1.2)	0.7		3.8 (1.0)	3.7 (1.4)	0.8	
Which observation on his bedside chart indicates a severe degree of dehydration?					81%	61%	0.1	

Which of the following medications AND indications is MOST appropriate:					92%	88%	0.9	
Which presentation is the most consistent with viral gastroenteritis?					27%	49%	0.1	
What investigations and management would you consider for this patient?					46%	35%	0.5	
What type and what rate of fluid should he be given?					46%	49%	0.99	
With regards to oral rehydration/feeding at home, which of the following pieces of advice should NOT be given to parents?					88%	90%	0.99	

MCQ, multiple choice questions; * p<0.05.

7 Supplementary Table 3: Long term (Week 8) retention of knowledge – MCQ results

	**Per Protocol Analysis**		
	**Traditional Tutorial**	**PlayMed Tutorial**	**p-value**
**Number of participants, n.**	8	21	
**Total MCQ Score (median (IQR) score out of 12)**	7.5 (6.5 – 8.5)	8 (7 – 9)	0.5
**Bronchiolitis MCQ Score (median (IQR) score out of 6)**	4.5 (3 – 6)	5 (4 – 5)	0.98
What is the most likely diagnosis?	75%	86%	0.9
What further features on history or examination is LEAST likely to indicate that admission is warranted?	50%	29%	0.5
Which of the following statements is incorrect about bronchiolitis?	100%	100%	#
Which of these statements is true about the management of bronchiolitis?	50%	62%	0.9
Which of the following clinical factors is least important when deciding whether an infant with bronchiolitis should be admitted to hospital?	63%	67%	0.99
When educating parents about bronchiolitis, which of the following pieces of advice should NOT be given?	100%	100%	#

**Gastroenteritis MCQ Score (median (IQR) score out of 6)**	3.5 (2 – 4.3)	4 (3 – 4)	0.5
Which observation on his bedside chart indicates a severe degree of dehydration?	75%	81%	0.99
Degree of weight loss is the most useful clinical indicator in order to estimate degree of dehydration.	38%	38%	0.99
Which presentation is the most consistent with viral gastroenteritis?	75%	67%	0.99
What investigations and management would you consider for this patient?	13%	38%	0.4
What type and what rate of fluid should he be given?	38%	48%	0.9
With regards to oral rehydration/feeding at home, which of the following pieces of advice should NOT be given to parents?	88%	100%	0.6

MCQ, multiple choice questions; # unable to calculate p-value; * p<0.05.

8 Supplementary Table 4: Long term (Week 8) reactions to educational experience

**Per Protocol Analysis**				
	**Traditional Tutorial**	**PlayMed Tutorial**	**p-value**	***d*** **(95% CI)**
**Number of participants, n.**	11	25		
**Bronchiolitis case specific reactions**				
The learning activity was beneficial for my paediatrics term.	78.4 (9.8)	72.1 (14.5)	0.2	
The learning activity motivated me to learn more about bronchiolitis.	74 (14.4)	71.3 (13.2)	0.6	
I was satisfied with the method in which my learning activity was conducted.	71.7 (14.8)	73.2 (14.8)	0.8	
I understand how to assess and manage a child with bronchiolitis.	74.7 (12.3)	70.8 (14.3)	0.4	
I am confident in my ability to assess and manage a child with bronchiolitis.	73.2 (13.7)	68.1 (68.1)	0.4	
In a child with bronchiolitis: I have an improved understanding of the key history and examination findings, and differential diagnoses.	75.1 (9.0)	72.6 (16.8)	0.6	
I have an improved understanding of ordering appropriate investigations.	75.1 (11.2)	73.5 (17.5)	0.7	
have an improved understanding of prescribing appropriate oxygen therapies.	66.9 (9.8)	68.8 (16.8)	0.7	
I have an improved understanding of how to manage fluid requirements and prescribe appropriate medications.	64.6 (16.8)	71.1 (13.9)	0.3	

I have an improved understanding of when an admission is required or when discharge is safe.	72.5 (9.3)	71.1 (14.9)	0.7	
I have an improved understanding of educating parents about bronchiolitis (in lay terms).	67.0 (23.6)	68.3 (16.8)	0.9	
The learning activity helped me prepare for, understand and deal with real-life clinical scenarios.	72.5 (9.4)	71.8 (17.8)	0.9	
The learning activity impacted on my paediatrics term.	64.7 (11.4)	65.4 (16.7)	0.9	
I am more confident in my ability to assess and manage gastroenteritis and dehydration because of my learning activities.	70.8 (9.2)	67.5 (16.8)	0.5	
I was able to apply the acquired knowledge from my learning activity in my paediatrics term.	74.4 (6.8)	55.3 (29.3)	0.01*	-0.76 (-1.60 – 0.08)
**Gastroenteritis case specific reactions**				
The learning activity was beneficial for my paediatrics term.	78.8 (10.9)	74.8 (15.4)	0.4	
The learning activity motivated me to learn more about gastroenteritis and dehydration.	75.6 (15.2)	70.8 (15.7)	0.4	
I was satisfied with the method in which my learning activity was conducted.	68.9 (24.0)	74.9 (12.7)	0.5	
I understand how to assess and manage a child with gastroenteritis and dehydration.	70.1 (13.8)	69.0 (19.4)	0.8	
I am confident in my ability to assess and manage a child with gastroenteritis and dehydration.	81.0 (65.0 – 82.0)	65.0 (60.0 – 76.5)	0.1	
I have an improved understanding of the key history and examination findings, and differential diagnoses.	72.2 (16.7)	69.3 (20.9)	0.7	
I have an improved understanding of assessing dehydration.	76.5 (13.9)	70.0 (21.0)	0.3	
I have an improved understanding of ordering appropriate investigations.	67.6 (23.9)	68.0 (20.2)	0.96	
I have an improved understanding of how to manage fluid requirements.	67.0 (17.5)	64.0 (21/3)	0.7	
I have an improved understanding of prescribing appropriate medications.	60.2 (23.3)	67.2 (22.2)	0.4	
I have an improved understanding of educating parents regarding oral rehydration at home.	81.0 (68.0 – 83.0)	75.0 (59.0 – 82.0)	0.6	
The learning activity helped me prepare for, understand and deal with real-life clinical scenarios.	72.6 (15.5)	69.2 (23.8)	0.6	
The learning activity impacted on my paediatrics term.	62.9 (18.6)	66.0 (23.0)	0.7	

I am more confident in my ability to assess and manage gastroenteritis and dehydration because of my learning activities.	72.0 (16.6)	66.0 (22.2)	0.4	
I was able to apply the acquired knowledge from my learning activity in my paediatrics term.	58.8 (18.0)	50.8 (28.4)	0.4	

Response data from survey questions recorded using a continuous interval scale (0 to 100) with pre-defined categories: 1 - 20: Strongly Disagree; 21 - 40: Disagree; 41 - 60: Neutral; 61 - 80: Agree; 81 - 100: Strongly Agree; 0: No response. * $p<0.05$.
