# Supplementary figures and images for: Serious games vs. traditional tutorials in the pandemic: a randomised controlled trial
Source: Front Med (Lausanne). 2024 Nov 21;11:1424024. doi: 10.3389/fmed.2024.1424024 (PMC11617181; doi:10.3389/fmed.2024.1424024)

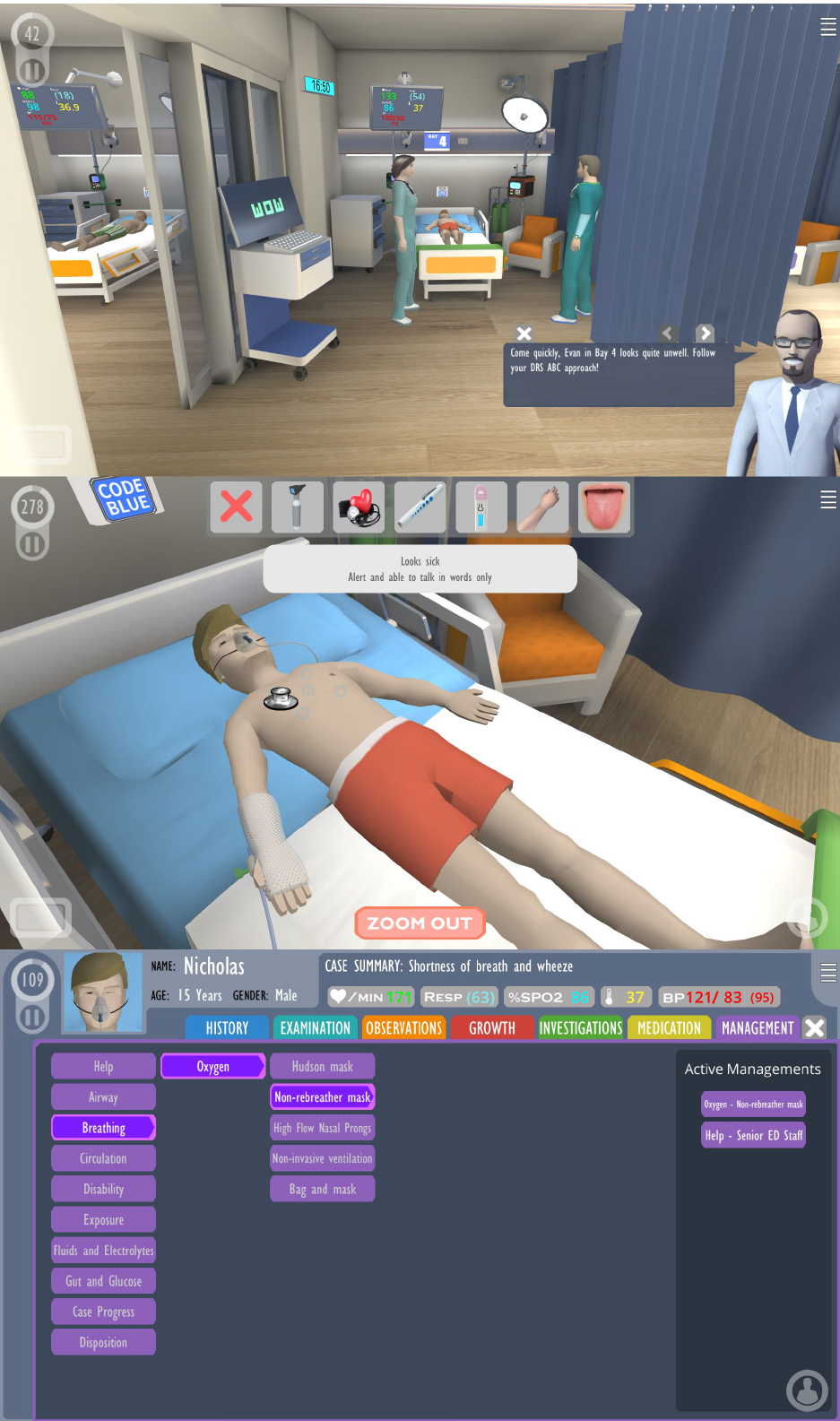

Supplement: Supplementary file 2 [file Image_1.TIF]
